# New Insights into the Regulation of Cell-Surface Signaling Activity Acquired from a Mutagenesis Screen of the *Pseudomonas putida* IutY Sigma/Anti-Sigma Factor

**DOI:** 10.3389/fmicb.2017.00747

**Published:** 2017-05-02

**Authors:** Karlijn C. Bastiaansen, Cristina Civantos, Wilbert Bitter, María A. Llamas

**Affiliations:** ^1^Department of Environmental Protection, Estación Experimental del Zaidín (CSIC)Granada, Spain; ^2^Section of Molecular Microbiology, Department of Molecular Cell Biology, VU University AmsterdamAmsterdam, Netherlands

**Keywords:** *Pseudomonas*, siderophore, iron, bacterial signal transduction, cell-surface signaling, gene regulation, proteolysis, sigma factor

## Abstract

Cell-surface signaling (CSS) is a signal transfer system that allows Gram-negative bacteria to detect environmental signals and generate a cytosolic response. These systems are composed of an outer membrane receptor that senses the inducing signal, an extracytoplasmic function sigma factor (σ^ECF^) that targets the cytosolic response by modifying gene expression and a cytoplasmic membrane anti-sigma factor that keeps the σ^ECF^ in an inactive state in the absence of the signal and transduces its presence from the outer membrane to the cytosol. Although CSS systems regulate bacterial processes as crucial as stress response, iron scavenging and virulence, the exact mechanisms that drive CSS are still not completely understood. Binding of the signal to the CSS receptor is known to trigger a signaling cascade that results in the regulated proteolysis of the anti-sigma factor and the activation of the σ^ECF^ in the cytosol. This study was carried out to generate new insights in the proteolytic activation of CSS σ^ECF^. We performed a random mutagenesis screen of the unique IutY protein of *Pseudomonas putida*, a protein that combines a cytosolic σ^ECF^ domain and a periplasmic anti-sigma factor domain in a single polypeptide. In response to the presence of an iron carrier, the siderophore aerobactin, in the extracellular medium, IutY is processed by two different proteases, Prc and RseP, which results in the release and activation of the σ^IutY^ domain. Our experiments show that all IutY mutant proteins that contain periplasmic residues depend on RseP for activation. In contrast, Prc is only required for mutant variants with a periplasmic domain longer than 50 amino acids, which indicates that the periplasmic region of IutY is trimmed down to ~50 amino acids creating the RseP substrate. Moreover, we have identified several conserved residues in the CSS anti-sigma factor family of which mutation leads to constitutive activation of their cognate σ^ECF^. These findings advance our knowledge on how CSS activity is regulated by the consecutive action of two proteases. Elucidation of the exact mechanism behind CSS activation will enable the development of strategies to block CSS in pathogenic bacteria.

## Introduction

Iron is an essential nutrient for virtually all life on earth, including bacteria. However, acquisition of iron from the environment and the host is greatly complicated due to the low bioavailability of this metal. To overcome this problem, most bacteria produce and secrete high-affinity iron chelating compounds called siderophores that can be recaptured by the cell through specific TonB-dependent receptors on the surface (Wandersman and Delepelaire, 2004). Fluorescent Pseudomonads such as *Pseudomonas aeruginosa* and *Pseudomonas putida* typically synthesize one main siderophore (e.g. pyoverdine) and some strains also a second lower-affinity siderophore. Nevertheless, they can produce up to 30 TonB-dependent receptors, which reflects the potential of these bacteria to use siderophores synthesized by other organisms (referred to as xeno- or heterologous-siderophores; Cornelis and Matthijs, 2002; Cornelis and Bodilis, 2009). Moreover, Pseudomonads have the ability to recognize other iron sources, including ferric citrate and host compounds such as heme or hemoglobin (Ochsner et al., 2000; Marshall et al., 2009; Cornelis and Dingemans, 2013). Expression of such a wide array of outer membrane receptors is an energetically costly process and is therefore tightly regulated (Andrews et al., 2003). Several TonB-dependent receptors are able to induce their own expression in response to the presence of their cognate iron source in the environment (Visca et al., 2002; Poole and McKay, 2003; Llamas et al., 2006) and this process is often regulated via a mechanism called cell-surface signaling (CSS) (Braun et al., 2006; Llamas et al., 2014). Importantly, these trans-envelope signal transduction cascades do not only regulate iron uptake but also bacterial competition and virulence processes (Aldon et al., 2000; Lamont et al., 2002; Llamas et al., 2008, 2009).

Typically, CSS systems consist of the outer membrane TonB-dependent receptor (also referred to as the CSS receptor), a transmembrane anti-sigma factor and a cytosolic extracytoplasmic function (ECF) sigma factor (σ^ECF^). CSS receptors form large 22-stranded β-barrels in the outer membrane and contain, in contrast to TonB-dependent receptors that are not involved in signal transduction, a periplasmic N-terminal extension referred to as the signaling domain (Koebnik, 2005). The signaling domain transduces the presence of the CSS stimulus to the anti-sigma factor and determines the specificity of the pathway (Noinaj et al., 2010). σ^ECF^ are part of the σ^70^ family of bacterial sigma factors, which are small and dissociable subunits of the bacterial RNA polymerase holoenzyme that are required for promoter recognition and transcription initiation (Paget and Helmann, 2003). σ^ECF^ are normally co-transcribed with their cognate inhibitor, the anti-sigma factor, which keeps the σ^ECF^ in an inactive state through an inhibitory interaction in the absence of the inducing stimulus. In Gram-negative bacteria, anti-sigma factors involved in CSS are generally cytoplasmic membrane proteins comprised of a cytosolic N-terminal tail (the N-tail) that binds the σ^ECF^, a single transmembrane segment, and a large periplasmic C-terminal domain that receives the signal from the CSS receptor (Llamas et al., 2014). Although the molecular mechanism of CSS is not yet completely understood, we and others have recently shown that in response to the inducing signal the CSS anti-sigma factor is subjected to a complex cascade of proteolytic cleavages (Draper et al., 2011; Bastiaansen et al., 2014, 2015a,b). This leads to the activation of the CSS σ^ECF^, which directs the RNAPc to the promoter region of its target genes, usually including the one coding for the cognate CSS receptor (Llamas et al., 2014).

**Figure 1:**
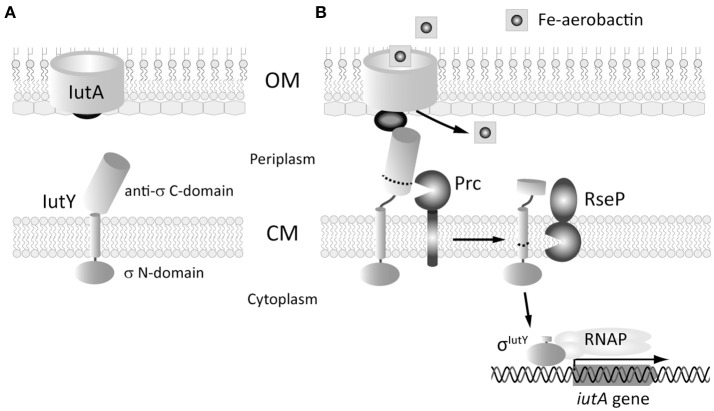
**Scheme of the proteolytic events that activate the aerobactin-induced Iut CSS system of ***P. putida***. (A)** The two proteins of the system, the IutA CSS receptor and the IutY sigma/anti-sigma hybrid protein are shown. In the absence of the siderophore, the sigma domain of IutY is inactive. **(B)** Binding of ferric-aerobactin to IutA generates a signal that, by analogy with other CSS systems, is likely transmitted to IutY by the signaling domain of IutA (dark gray ball). This triggers the proteolytic cleavage of the anti-sigma C-domain of IutY by the sequential action of the Prc and the RseP proteases. The resulting σ^IutY^ protein domain can then interact with the RNAP and initiates expression of the *iutA* gene. OM, outer membrane; CM, cytoplasmic membrane. Adapted from Bastiaansen et al. (2014) and Llamas et al. (2014).

We have recently described an unusual CSS system in the saprophyte bacterium *P. putida*, the Iut system, which is employed by the bacterium to regulate the uptake of aerobactin, a siderophore produced by certain *E. coli* species (Bastiaansen et al., 2014). The CSS receptor of this system is encoded by the *iutA* gene and displays all the typical characteristics. However, the adjacent *iutY* gene codes for a unique hybrid protein, which contains both a cytosolic σ^ECF^ domain (σ^IutY^) and a periplasmic anti-sigma factor domain that are separated by a single transmembrane segment (Bastiaansen et al., 2014). We have demonstrated that upon activation of the system the IutY protein is subjected to regulated intramembrane proteolysis (RIP) in order to liberate and activate the cytosolic σ^IutY^ domain (Bastiaansen et al., 2014; Figure 1). In the presence of aerobactin the periplasmic anti-sigma domain of IutY is physically removed through the sequential action of at least two proteases; the C-terminal processing protease Prc, which likely acts in the periplasm, and the transmembrane site-2 metalloprotease RseP, which cleaves IutY in or near the transmembrane segment. This results in the generation of an N-terminal fragment of ~23 kDa that represents the σ^IutY^ domain (Bastiaansen et al., 2014). Deletion of either Prc or RseP completely abolishes aerobactin-induced σ^IutY^ activity, and significantly impairs activation of classical CSS pathways in which the sigma and the anti-sigma factors are two separate proteins (Bastiaansen et al., 2014), highlighting the importance of both proteases in CSS regulation. To get more insight into the (proteolytic) activation of CSS, we used the hybrid IutY protein as a model system and performed a random mutagenesis to select for constitutively active IutY mutants that did not require the presence of the signal for activation. Analysis of these proteins in different genetic backgrounds has allowed us to identify factors that determine dependency on Prc and/or RseP, as well as several conserved residues within the CSS anti-sigma factor family that are crucial for the proper control of the CSS cascade. These results are not always consistent with the mechanisms currently described in literature, thus providing new avenues to explore in further studies and novel insights to deregulate these important bacterial regulatory circuits.

## Materials and methods

### Bacterial strains and growth conditions

Strains used in this study are listed in Table 1. *P. putida* was routinely grown in liquid LB (Sambrook et al., 1989) at 30°C on a rotary shaker operated at 200 rpm. For IutY induction experiments, *P. putida* cells were grown in liquid CAS medium (Llamas et al., 2006) containing 200 μM of 2,2′-bipyridyl (iron-restricted conditions) and 1 mM isopropyl β-D-1-thiogalactopyranoside (IPTG) supplemented with aerobactin containing supernatant derived from iron-restricted cultures of *E. coli* C600 (ColV-K30) in 1:1 proportion (Bastiaansen et al., 2014). For non-inducing conditions, the supernatant of iron-restricted cultures of *E. coli* C600 was used. Induction experiments with ferrioxamine and ferrichrome were performed following *P. putida* or *P. aeruginosa* growth in CAS medium supplemented with 400 μM of 2,2′-bipyridyl and in the absence or presence of 1 μM iron-free ferrioxamine B or 40 μM iron-free ferrichrome (both purchased from Sigma-Aldrich). Construction of null mutants was performed by allelic exchange using the suicide vector pKNG101 as previously described (Bastiaansen et al., 2014). Mutants were verified by PCR. When necessary, antibiotics were used at the following final concentrations (μg ml^−1^): ampicillin (Ap), 100; piperacillin (Pip), 25; streptomycin (Sm), 50 for *E. coli* and 100 for *Pseudomonas*; tetracycline (Tc), 12.5 for *E. coli* and 20 for *Pseudomonas*.

**Table 1:** **Bacterial strains and plasmids used in this study^**a**^**.

**Strain**	**Characteristics**	**References**
***E. coli***		
DH5α	*supE44 Δ*(l*acZYA-argF*)*U169* ϕ80 *lacZ*ΔM15 *hsdR17* (${\text{r}}_{\text{K}}^{-}$ ${\text{m}}_{\text{K}}^{+}$) *recA1 endA1 gyrA96 thi1 relA1*; Nal^R^	Hanahan, 1983
TOP10F′	F′[*lacI^*q*^, Tn10* (TetR)] *mcrA* Δ(*mrr-hsdRMS-mcrBC*) Φ80*lacZ*ΔM15 Δ*lacX74 recA1 araD139* Δ(*ara leu*) 7697 *galU galK rpsL* (StrR) *endA1 nupG*; Tc^R^	Invitrogen
***P. putida***		
KT2440	*hsdR*1, wild-type strain; Rif^R^	Franklin et al., 1981
Δ*iutY*	Markerless KT2440 null mutant in the *iutY* (PP2192) gene; Rif^R^	Bastiaansen et al., 2014
Δ*prc*	Markerless KT2440 null mutant in the *prc* (PP1719) gene; Rif^R^	Bastiaansen et al., 2014
Δ*rseP*	Markerless KT2440 null mutant in the *rseP* (PP1598) gene; Rif^R^	Bastiaansen et al., 2014
Δ*iutA*	Markerless KT2440 null mutant in the *iutA* (PP2193) gene; Rif^R^	This study
Δ*iutY* Δ*prc*	Δ*iutY* carrying an additional markerless null mutation in *prc* (PP1719) gene; Rif^R^	This study
Δ*iutY* Δ*rseP*	Δ*iutY* carrying an additional markerless null mutation in *rseP* (PP1598) gene; Rif^R^	This study
Δ*iutY* Δ*iutA*	Δ*iutY* carrying an additional markerless null mutation in *iutA* (PP2193) gene; Rif^R^	This study
***P. aeruginosa***		
PAO1 *pvdF* Δ*foxR*	PAO1 *pvdF* with a deletion of amino acids 12–295 of the FoxR (PA2467) protein; Km^R^	Mettrick and Lamont, 2009
PAO1 *pvdF* Δ*fiuR*	PAO1 *pvdF* with a deletion of amino acids 12–319 of the FiuR (PA0471) protein; Km^R^	Mettrick and Lamont, 2009
**PLASMID**		
pKNG101	Gene replacement suicide vector, *ori*R6K, *oriT*RK2, *sacB*; Sm^R^	Kaniga et al., 1991
pKΔprc	pKNG101 carrying the *P. putida prc* (PP1719) deletion construct; Sm^R^	Bastiaansen et al., 2014
pKΔrseP	pKNG101 carrying the *P. putida rseP* (PP1598) deletion construct; Sm^R^	Bastiaansen et al., 2014
pKΔiutA	pKNG101 carrying in XbaI-BamHI a 2.8-Kb PCR fragment containing the regions up- and downstream the *P. putida iutA* (PP2193) gene; Sm^R^	This study
pMMB67EH	IncQ broad-host range plasmid, *lacI^*q*^*; Ap^R^	Fürste et al., 1986
pMMBK1-HA	pMMB67EH carrying an N-terminally HA-tagged *P. putida iutY* gene (PP2192); Ap^R^	Bastiaansen et al., 2014
pMMBK1-HA-168	pMMBK1-HA in which a premature stop codon was inserted after residue 168 (from the random mutagenesis screen); Ap^R^	This study
pMMBK1-HA-201	pMMBK1-HA in which a premature stop codon was inserted after residue 201 (from the random mutagenesis screen); Ap^R^	This study
pMMBK1-HA-260	pMMBK1-HA in which a premature stop codon was inserted after residue 260 (from the random mutagenesis screen); Ap^R^	This study
pMMBK1-HA-318	pMMBK1-HA in which a premature stop codon was inserted after residue 318 (from the random mutagenesis screen); Ap^R^	This study
pMMBK1-HA-365	pMMBK1-HA in which a premature stop codon was inserted after residue 365 (from the random mutagenesis screen); Ap^R^	This study
pMMBK1-HA-225	pMMB67EH carrying in EcoRI-HindIII a 6.75-Kb PCR fragment encoding the first 225 amino acids of the *P. putida* IutY protein with an N-terminal HA-tag; Ap^R^	This study
pMMBK1-HA-236	pMMB67EH carrying in EcoRI-HindIII a 7.08-Kb PCR fragment encoding the first 236 amino acids of the *P. putida* IutY protein with an N-terminal HA-tag; Ap^R^	This study
pMMBK1-HA-293	pMMB67EH carrying in EcoRI-HindIII a 8.79-Kb PCR fragment encoding the first 293 amino acids of the *P. putida* IutY protein with an N-terminal HA-tag; Ap^R^	This study
pMMBK1-HA-338	pMMB67EH carrying in EcoRI-HindIII a 1.01-Kb PCR fragment encoding the first 338 amino acids of the *P. putida* IutY protein with an N-terminal HA-tag; Ap^R^	This study
pMMBK1-HA-G201C	pMMBK1-HA in which glycine-201 has been mutated to a cysteine; Ap^R^	This study
pMMBK1-HA-D210A	pMMBK1-HA in which aspartic acid-210 has been mutated to an alanine; Ap^R^	This study
pMMBK1-HA-F226L	pMMBK1-HA in which phenylalanine-226 has been mutated to a leucine; Ap^R^	This study
pMMBK1-HA-E230K	pMMBK1-HA in which glutamic acid-230 has been mutated to a lysine; Ap^R^	This study
pMMBK1-HA-R231Q	pMMBK1-HA in which arginine-231 has been mutated to a glutamine; Ap^R^	This study
pMMBK1-HA-A240E	pMMBK1-HA in which alanine-240 has been mutated to a glutamic acid; Ap^R^	This study
pMMBK1-HA-F251S	pMMBK1-HA in which phenylalanine-251 has been mutated to a serine; Ap^R^	This study
pMMBK1-HA-V253D	pMMBK1-HA in which valine-253 has been mutated to an aspartic acid; Ap^R^	This study
pMMBK1-HA-R271C	pMMBK1-HA in which arginine-271 has been mutated to a cysteine; Ap^R^	This study
pMMBK1-HA-W301G	pMMBK1-HA in which tryptophan-301 has been mutated to a glycine; Ap^R^	This study
pMMBK1-HA-G304D	pMMBK1-HA in which glycine-304 has been mutated to an aspartic acid; Ap^R^	This study
pMMBK1-HA-T365I	pMMBK1-HA in which threonine-365 has been mutated to an isoleucine; Ap^R^	This study
pMMBK1-HA-LtoP	pMMBK1-HA in which leucine-313, leucine-317 and leucine-320 have been mutated to prolines; Ap^R^	This study
pMMB/HA-FoxR	pMMB67EH carrying the *P. aeruginosa foxR* gene (PA2467) which has been N-terminally HA-tagged; Ap^R^	Bastiaansen et al., 2015a
pMMB/HA-FoxR-D132A	pMMB/HA-FoxR in which aspartic acid-132 has been mutated to an alanine; Ap^R^	This study
pMMB/HA-FoxR-R153Q	pMMB/HA-FoxR in which asparagine-153 has been mutated to a glutamine; Ap^R^	This study
pMMB/HA-FoxR-V180D	pMMB/HA-FoxR in which valine-180 has been mutated to an aspartic acid; Ap^R^	This study
pMMB/HA-FiuR	pMMB67EH carrying in EcoRI-XbaI a 0.97-Kb PCR fragment containing a N-terminally HA-tagged *P. aeruginosa fiuR* gene; Ap^R^	Bastiaansen et al., 2015a
pMMB/HA-FiuR-D133A	pMMB/HA-FiuR in which aspartic acid-133 has been mutated to an alanine; Ap^R^	This study
pMMB/HA-FiuR-R154Q	pMMB/HA-FiuR in which asparagine-154 has been mutated to a glutamine; Ap^R^	This study
pMMB/HA-FiuR-V177D	pMMB/HA-FiuR in which valine-177 has been mutated to an aspartic acid; Ap^R^	This study
pMP220	IncP broad-host-range *lacZ* fusion vector; Tc^R^	Spaink et al., 1987
pMPK4	pMP220 carrying the *P. putida iutA* (PP2193) promoter region cloned upstream the *lacZ* gene; Tc^R^	Bastiaansen et al., 2014
pMPR8b	pMP220 carrying the *P. aeruginosa foxA* (PA2466) promoter region cloned upstream the *lacZ* gene; Tc^R^	Llamas et al., 2006
pMPFiuA	pMP220 carrying the *P. aeruginosa fiuA* (PA0470) promoter region cloned upstream the *lacZ* gene; Tc^R^	Llamas et al., 2006

a *Ap^R^, Km^R^, Nal^R^, Rif^R^, Sm^R^, and Tc^R^, resistance to ampicillin, kanamycin, nalidixic acid, rifampicin, streptomycin, and tetracycline, respectively*.

### Plasmid construction and molecular biology

Plasmids used are listed in Table 1 and primer sequences in Table S1. Phusion® Hot Start High-Fidelity DNA Polymerase (New England BioLabs) was used for PCR amplifications. Point mutations were introduced by nested PCR. All constructs were verified by DNA sequencing and transferred to *P. putida* or *P. aeruginosa* by electroporation (Choi et al., 2006).

### Random mutagenesis of IutY

Random mutagenesis of the entire N-terminally HA-tagged *iutY* gene was performed by error prone PCR on the pMMBK1-HA plasmid (Bastiaansen et al., 2014) using the low fidelity polymerase Pfu-Pol (exo^−^) D473G^*^ (Biles and Connolly, 2004) with primers NHA-PP2192-E and PP2192R-X (Table S1). PCR products were subsequently cloned in the EcoRI-XbaI restriction sites of the pMMB67EH plasmid (Fürste et al., 1986). After transformation to *E. coli* TOP10F' all clones were pooled prior to the isolation of plasmid DNA and transferred to *P. putida* KT2440 bearing the pMPK4 plasmid by electroporation (Choi et al., 2006). Clones with high β-galactosidase activity were selected on LB plates containing 50 μg/ml 5-bromo-4-chloro-3-indolyl-beta-D-galacto-pyranoside (X-gal) and 1 mM IPTG and sequenced following plasmid DNA isolation. For the second round of the screen, mutations in the cytosolic sigma factor domain of IutY were excluded by using the presence of a unique SacII restriction site at the position of the transmembrane domain of *iutY*. The pool of randomly mutagenized PCR products were cloned as SacII-XbaI fragments, thus containing only the periplasmic region of the protein, in the corresponding sites of the pMMBK1-HA plasmid, thereby keeping the HA-tagged cytosolic domain of *iutY* contained in this plasmid intact.

### Enzyme assay

β-Galactosidase measurements were performed in soluble cell extracts using the *o*-nitrophenyl-β-D-galactopyranoside (ONPG) substrate (Sigma-Aldrich) as previously described (Llamas et al., 2006). Activity is expressed in Miller units. Each assay was run in duplicate at least three times and the data given are the average with error bars in all graphs indicating standard deviation (*SD*).

### SDS-PAGE and western-blot

*P. putida* was grown to late log-phase in CAS medium containing 200 μM of 2,2′-bipyridyl and 1 mM IPTG, in the presence of either aerobactin deficient or aerobactin containing supernatant. Subsequently, cells were pelleted by centrifugation and heated for 10 min at 95°C following solubilization in SDS-PAGE sample buffer. Sample normalization was done according to the OD_660_ of the bacterial culture. Proteins were separated by SDS-PAGE containing 15% (w/v) acrylamide and electrotransferred to nitrocellulose membranes (Millipore). As a loading control staining with a Ponceau S dye (Serva) was performed. Subsequently, HA-tagged proteins were detected using a monoclonal antibody directed against the influenza hemagglutinin epitope (HA.11, Covance) as previously described (Bastiaansen et al., 2014).

### Computer-assisted analyses

*P. putida* and *P. aeruginosa* genome sequences were obtained at www.pseudomonas.com (Winsor et al., 2011). Sequence alignments were performed with ClustalW (Goujon et al., 2010). Tertiary structure prediction was performed with the Phyre2 program at www.sbg.bio.ic.ac.uk/~phyre2 (Kelley and Sternberg, 2009). The quality of this prediction was assessed using DALI (Holm and Rosenstrom, 2010) and TM-align (Zhang and Skolnick, 2005), which calculate the root-mean-square deviation (RMSD) of the model by comparing it with its structure template.

## Results

### Removing (part of) the anti-sigma domain of IutY activates σ^IutY^ independent of aerobactin

**Figure 2:**
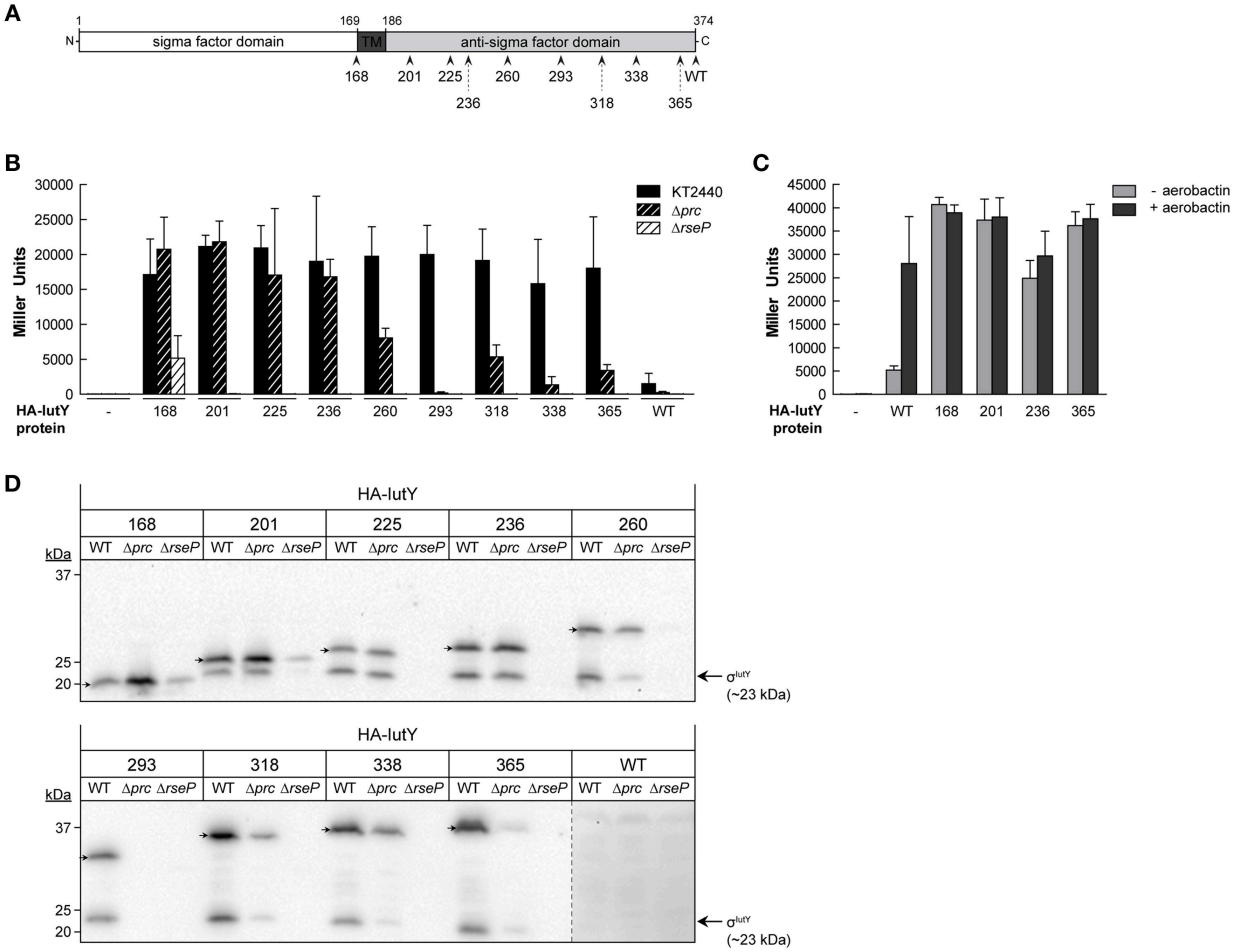
**Activity and processing of constitutively active truncated IutY variants. (A)** Schematic representation of the *P. putida* sigma/anti-sigma hybrid protein IutY drawn to scale. The cytosolic sigma factor domain, transmembrane (TM) domain, and periplasmic anti-sigma factor domain are shown. The number of amino acids of the various truncated variants tested are indicated. **(B)** β-Galactosidase activity of the *P. putida* KT2440 wild-type strain (black bars) and its isogenic Δ*prc* (black striped bars) or Δ*rseP* (white striped bars) mutants bearing the transcriptional fusion *iutA*::*lacZ* (pMPK4 plasmid) and a pMMB67EH-derivate expressing one of the indicated truncated IutY variants grown in LB supplemented with 1 mM IPTG. **(C)** β-Galactosidase activity of the *P. putida* Δ*iutY* mutant bearing the transcriptional fusion *iutA*::*lacZ* (pMPK4 plasmid) and the pMMB67EH (empty), pMMBK1-HA (WT), pMMBK1-HA-168, -201, -236, or -365 construct expressing one of the IutY variants grown in low iron medium supplemented with 1 mM IPTG in the absence (light gray bars) or presence (dark gray bars) of aerobactin. **(D)** The *P. putida* KT2440 wild-type strain (WT) and its isogenic Δ*prc* or Δ*rseP* mutant expressing one of the indicated truncated IutY variants were grown in LB supplemented with 1 mM IPTG. Proteins were detected by Western-blot using an anti-HA antibody. Position of the full-length truncate protein is indicated by an arrow. The protein band corresponding to the σ^IutY^ domain and the molecular size marker (in kDa) are also indicated. Vertical dotted line indicates combination of two separate images to show prolonged exposure of the HA-IutY wild-type protein blot, which was not visible in this condition.

To identify residues in the IutY protein important for activation of the cytosolic σ^IutY^ domain, we performed a random mutagenesis of the entire *iutY* gene and selected for gain of function mutations. An N-terminally HA-tagged version of *iutY*, which encodes a protein with similar activity as the non-tagged variant (Bastiaansen et al., 2014), was mutagenized by error prone PCR. The resulting products were cloned in the pMMB67EH vector behind an IPTG-inducible P*tac* promoter and the pool of plasmids was transferred to the *P. putida* KT2440 wild-type strain bearing the σ^IutY^-dependent *iutA*::*lacZ* transcriptional fusion (Bastiaansen et al., 2014). Subsequently, a blue-white screening was performed on solid LB medium supplemented with X-gal. Activity of σ^IutY^ induces expression from the *iutA* promoter and therefore increases *lacZ* production, resulting in blue colonies. A total of 46 bright blue colonies were selected. Sequence analyses showed that only seven of the blue clones encoded a full-length IutY protein, while the majority (39) acquired a premature stop codon in the *iutY* gene and therefore produced truncated IutY variants. This indicates that the most efficient method to create constitutive activity of IutY is to introduce a premature stop codon and these mutations will be discussed first. The shortest truncated variant (until amino acid 168) contained only the cytosolic σ^IutY^ domain, while the longest truncated variant obtained (until amino acid 365) only lacked the last nine amino acids of the protein (selected mutants are shown in Figure 2A and Figure S1). Other premature stop codons were randomly distributed over the periplasmic domain of the protein, suggesting that IutY can be truncated at any position in the anti-sigma domain to produce a constitutively active σ^IutY^. Some of the truncated IutY variants also contained point mutations in the N-terminal IutY sigma factor domain. Although it was unlikely that these mutations were the cause of the increased σ^IutY^ activity, we exchanged the N-terminus of several truncated IutY variants for the wild-type sequence to exclude any effect of alterations in the sigma domain.

To validate the results of the screen and quantify σ^IutY^ activity, we measured β-galactosidase activity of the *P. putida* wild-type strain bearing the *iutA*::*lacZ* fusion upon expression of five selected IutY truncated variants (HA-IutY-168, -201, -260, -318, and -365). We also included in this analysis four additional IutY constructs, encoding HA-IutY-225, -236, -293, or -338 (Table 1). This resulted in a total of nine truncated variants of different length, covering the complete periplasmic anti-sigma domain of IutY (Figure 2A and Figure S1). Activity of these proteins was compared with that of the full-length IutY protein upon growth of *P. putida* in LB medium. All strains expressing a truncated IutY protein displayed very high *lacZ* activity (i.e., high *iutA* promoter activity; Figure 2B). This suggests that removing (part of) the periplasmic anti-sigma domain of IutY strongly triggers σ^IutY^ activity in a signal-independent manner. To confirm this, the HA-IutY-168, -201, -236, and -365 truncated variants (Figure 2A) were expressed in a Δ*iutY* deletion mutant (Bastiaansen et al., 2014) and σ^IutY^ activity was measured in the absence and presence of aerobactin. As expected, addition of the siderophore to the medium significantly increased expression from the *iutA* promoter in the strain expressing the full-length HA-IutY protein (~5-fold; Figure 2C, WT protein). All four tested truncated IutY variants exhibited very high promoter activity in the absence of aerobactin, which did not increase upon addition of the siderophore (Figure 2C). The level of *lacZ* activity produced by the mutant proteins was similar to that obtained with the wild-type protein upon induction with aerobactin. This indicates that removing part of the C-terminal region of IutY is by itself sufficient to produce maximal σ^IutY^ activity.

### Role of the Prc and RseP proteases in the activation of the truncated IutY variants

IutY is known to be processed by both the Prc and RseP proteases in response to aerobactin, which allows the release and activation of the cytosolic σ^IutY^ domain (~23 kDa; Figure 1; Bastiaansen et al., 2014). To assay whether the activity of the truncated IutY variants still depends on these proteases, σ^IutY^ activity was tested by measuring *iutA*::*lacZ* expression of the truncated *iutY* variants in Δ*prc* and Δ*rseP* mutant strains (Bastiaansen et al., 2014) upon growth in LB (Figure 2B). The presence of an N-terminal HA-tag in the truncated variants allowed us to also examine protein processing by Western-blot (Figure 2D). These analyses showed that the activity of all truncated IutY variants, except that of the IutY-168 protein, was completely dependent on the RseP protease (Figure 2B). RseP is known to generate the active σ^IutY^ domain by cleaving within or near the transmembrane domain (Bastiaansen et al., 2014), a domain that is lacking in IutY-168 (Figure 2A). In accordance, two protein bands corresponding to the predicted size of the full-length truncated variant and the σ^IutY^ domain, respectively, were visible for all constructs except IutY-168 (Figure 2D). For IutY-168, only the band that corresponds to the predicted full-length size of the protein was detected (Figure 2D). However, although IutY-168 is not processed by RseP, its activity was significantly reduced in the Δ*rseP* mutant when compared with the wild-type strain (Figure 2B). This may be related with a reduced stability of IutY-168 in the absence of RseP, since the amount of this protein in this mutant was significantly lower (Figure 2D). Surprisingly, the other truncated proteins were not detected in the Δ*rseP* mutant (Figure 2D), which could suggest a role for RseP in the assembly and/or stability of these proteins in the membrane.

The β-galactosidase measurements in the Δ*prc* mutant showed that the activity of the four shortest truncated variants (IutY-168, -201, -225, and -236) was not affected by the deletion of Prc, while the activity of the longer truncated variants was either completely abolished (IutY-293) or significantly reduced (IutY-260, -318, -338, and -365; Figure 2B). Western-blot analyses revealed that the stability and processing of the four truncated variants that were independent of Prc were not affected, whereas clear differences were observed for the other truncated variants between the wild-type background and the Δ*prc* mutant (Figure 2D). Importantly, stability of the IutY proteins in the Δ*prc* mutant correlated with activity. The protein levels of the truncated variants with impaired activity in this protease mutant (namely IutY-260, -293, -318, -338, and -365) were significantly lower than that of the fully active proteins (IutY-168, -201, -225, and -236; Figures 2B,D). Altogether, our results indicate that IutY proteins shorter than 236 amino acids are probably not processed by Prc and likely become direct substrates of the RseP protease.

### Single amino acid substitutions in the anti-sigma domain of IutY can activate σ^IutY^ independent of aerobactin

**Figure 3:**
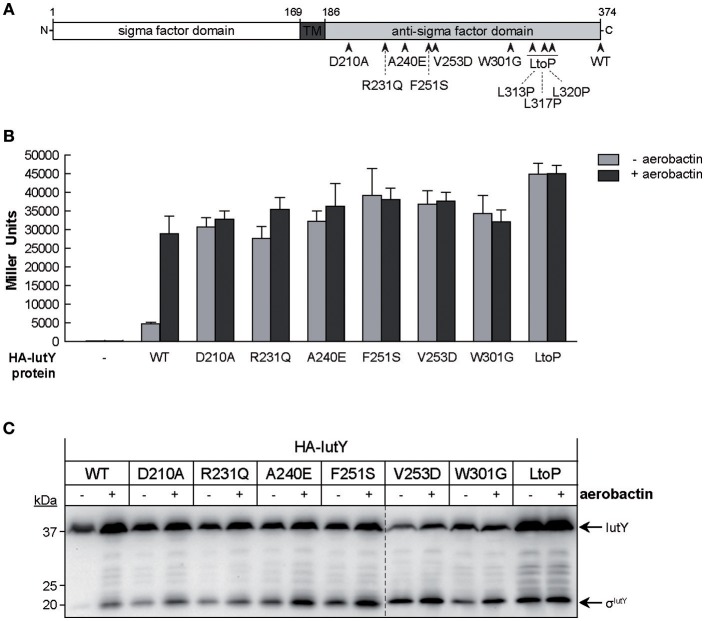
**Activity and processing of constitutively active IutY point mutants. (A)** Schematic representation of the *P. putida* IutY protein drawn as in Figure 2A. The position of the IutY single mutations tested is indicated. **(B)** β-Galactosidase activity of the *P. putida* Δ*iutY* mutant strain bearing the transcriptional fusion *iutA*::*lacZ* (pMPK4 plasmid) and the pMMB67EH (empty, -), pMMBK1-HA (WT), pMMBK1-HA-D210, -R231Q, -A240E, -F251S, -V253D, -W301G, or -LtoP construct expressing the corresponding IutY protein variant grown in low iron medium supplemented with 1 mM IPTG in the absence (light gray bars) or presence (dark gray bars) of aerobactin. **(C)** The *P. putida* Δ*iutY* strain expressing the indicated N-terminally HA-tagged IutY mutant protein was grown in the absence (–) or presence (+) of aerobactin. Proteins were detected by Western-blot using an anti-HA antibody. Position of the protein fragments (full length IutY and σ^IutY^ domain) and the molecular size marker (in kDa) are indicated.

In addition to the truncated IutY variants, we obtained several clones encoding full-length IutY protein variants in the random mutagenesis screen. Most of these mutants contained multiple mutations (between 2 and 4), both in the periplasmic anti-sigma domain and the cytosolic σ^IutY^ domain (data not shown). In order to avoid amino acid substitutions in the cytosolic region of the protein, we repeated the screen but with a different cloning strategy to exclude mutations in σ^IutY^ (described in Section Material and Methods). Subsequently, the plasmids were transferred to *P. putida* KT2440 and constitutive σ^IutY^ activity was detected on LB plates supplemented with X-gal. Bright blue clones were first analyzed by Western-blot to select for strains expressing a complete IutY protein and then sequenced. This approach identified in total 20 full-length active IutY mutants (Figure S2). Two of these clones contained only a single changed residue in the anti-sigma domain (R231Q and W301G), while the others contained several mutations. We selected 11 positions that were affected in several IutY mutant proteins (Figure S2) and introduced these single amino acid substitutions in the wild-type N-terminally HA-tagged *iutY* gene. Moreover, we generated two additional mutants in conserved residues (Figure S2). The functionality of these 13 IutY mutant proteins was tested by determining β-galactosidase activity of the *iutA*::*lacZ* fusion upon expression in the Δ*iutY* mutant in the absence or presence of aerobactin. Activity of six IutY variants was similar to that of the wild-type IutY protein (data not shown), indicating that these single mutations did not confer constitutive activity to the protein. In contrast, the activity of the IutY-D210A, -R231Q, -A240E, -F251S, -V253D, -W301G, and -LtoP (in which three leucines that form a conserved motif implicated in the interaction with the CSS receptor (Enz et al., 2003) were mutated to proline residues) proteins (Figure 3A) was significantly increased in the absence of aerobactin (Figure 3B). Activity of these mutant proteins did not further increase upon addition of the siderophore (Figure 3B), which indicates that these IutY protein variants completely lost their capacity to respond to the inducing signal. The production of the σ^IutY^ domain was analyzed by Western-blot (Figure 3C). It should be noted that the wild-type (WT) protein was detected in this analysis in which the strains were grown in low iron conditions (Figure 3C), whereas the same protein could not be detected after growth in LB medium (Figure 2D). This is likely due to a higher stability of IutY in iron limitation, which is not surprising given that iron limitation is required *in vivo* for expression and activation of CSS σ^ECF^/anti-sigma factor pairs that regulate iron uptake (Llamas et al., 2014). While in the strain expressing the wild-type IutY protein the σ^IutY^ domain (~23 kDa) was detected in high amounts only upon growth with aerobactin, it was highly present in all constitutively active mutants even in absence of the siderophore (Figure 3C) and correlated well with the measured activity of the proteins (Figure 3B). Together, these results show that the constitutively active IutY mutants are, as the wild-type protein, activated by the proteolytic release of the σ^IutY^ domain.

### Role of the Prc and RseP proteases, and the IutA CSS receptor in the activation of constitutively active IutY point mutants

**Figure 4:**
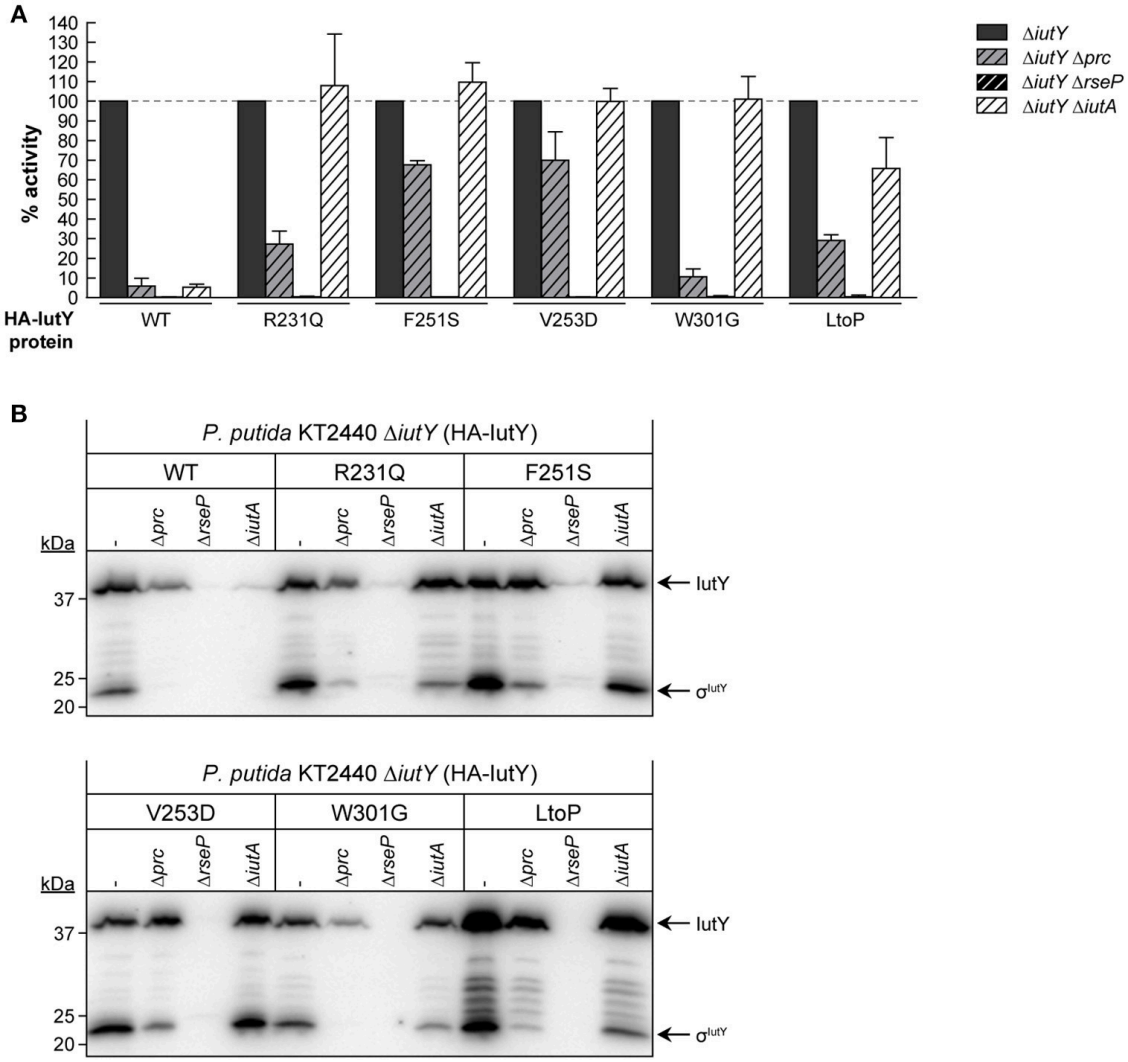
**Dependency of constitutively active IutY point mutants on Prc, RseP, and IutA. (A)** β-Galactosidase activity of the *P. putida* Δ*iutY*, Δ*iutY* Δ*prc*, Δ*iutY* Δ*rseP*, and Δ*iutY* Δ*iutA* strains bearing the transcriptional fusion *iutA*::*lacZ* (pMPK4 plasmid) and the pMMBK1-HA (WT), pMMBK1-HA-R231Q, -F251S, -V253D, -W301G, or -LtoP construct expressing one of the IutY variants and grown in aerobactin-contained low iron medium with 1 mM IPTG. In each separate experiment the activity in the Δ*iutY* strain has been set to 100%. **(B)** The same strains were grown as above and the IutY proteins were detected by Western-blot using an anti-HA antibody. Position of the protein fragments (full length IutY and σ^IutY^ domain) and the molecular size marker (in kDa) are indicated.

To examine whether RseP and Prc are required for the proteolytic activation of the IutY full-length mutant proteins (Figure 3A), we measured *iutA*::*lacZ* activity in Δ*iutY* Δ*prc* and Δ*iutY* Δ*rseP* double mutants upon bacterial growth in aerobactin. Similar to the wild-type protein, activity of all mutant proteins was completely abolished in the Δ*iutY* Δ*rseP* mutant (Figure 4A), indicating that the RseP protease is essential for the activation of these proteins. Prc deletion is known to completely abolish activation of IutY when this protein is produced from the bacterial chromosome (Bastiaansen et al., 2014). Interestingly, activity of wild-type IutY expressed from plasmid in the absence of Prc (Δ*iutY* Δ*prc* mutant) was considerably reduced but not completely abrogated, since the protein retained ~7% of the *lacZ* expression measured in the Δ*iutY* single mutant (Figure 4A). Therefore, this result suggests that overexpression of *iutY* from plasmid produces protein levels that can bypass the absolute requirement for Prc. Similar to the wild-type protein, the IutY-W301G mutant also retained ~10% of its activity in the Δ*iutY* Δ*prc* strain, whereas the IutY-R231Q and -LtoP proteins retained ~30% of their activity (Figure 4A). This implies that these proteins are mostly dependent on Prc for activation. In contrast, activation of the IutY-F251S and -V253D proteins was largely independent of Prc, since ~70% of their activity was retained in the protease mutant (Figure 4A). Western-blot analyses showed that all IutY proteins were notably less stable in conditions in which they were not or only partially active (Figure 4B). In accordance, all proteins were hardly detectable in the Δ*iutY* Δ*rseP* mutant (Figure 4B), in which activity is completely abolished (Figure 4A). In the Δ*iutY* Δ*prc* mutant, both the full-length IutY form and the σ^IutY^ domain were clearly detectable for those IutY variants that were active in this mutant (IutY-F251S and -V253D), only low amounts were detectable for those in which activity was compromised (IutY-R231Q and -LtoP), and they were hardly detectable for proteins with impaired activity (wild-type IutY and IutY-W301G; Figure 4B).

Importantly, our results show that single amino acid substitutions in the periplasmic anti-sigma domain of IutY can lead to constitutively active proteins that require RseP and/or Prc for activation, but not the presence of aerobactin. Since the signal in CSS pathways is sensed by the CSS outer membrane receptor (Llamas et al., 2014), this suggests that the IutY mutant proteins do not depend on IutA. To test this, a Δ*iutY* Δ*iutA* double mutant was constructed and the activity of the mutant proteins in response to aerobactin was assayed. As expected, deletion of *iutA* resulted in only residual activity of the wild-type IutY protein, with only ~5% of the *lacZ* production (Figure 4A; Bastiaansen et al., 2014). In contrast, all tested IutY mutants (R231Q, F251S, V253D, W301G, and LtoP) were active in the Δ*iutY* Δ*iutA* double mutant (Figure 4A). In agreement with these results, Western-blot analyses showed that only the amount of the wild-type IutY protein was significantly affected in the absence of IutA (Figure 4B). This confirms that the activity of the IutY mutant proteins does no longer depend on the IutA receptor.

### Generation of constitutively active FoxR and FiuR anti-sigma factor proteins by mutating conserved periplasmic residues

**Figure 5:**
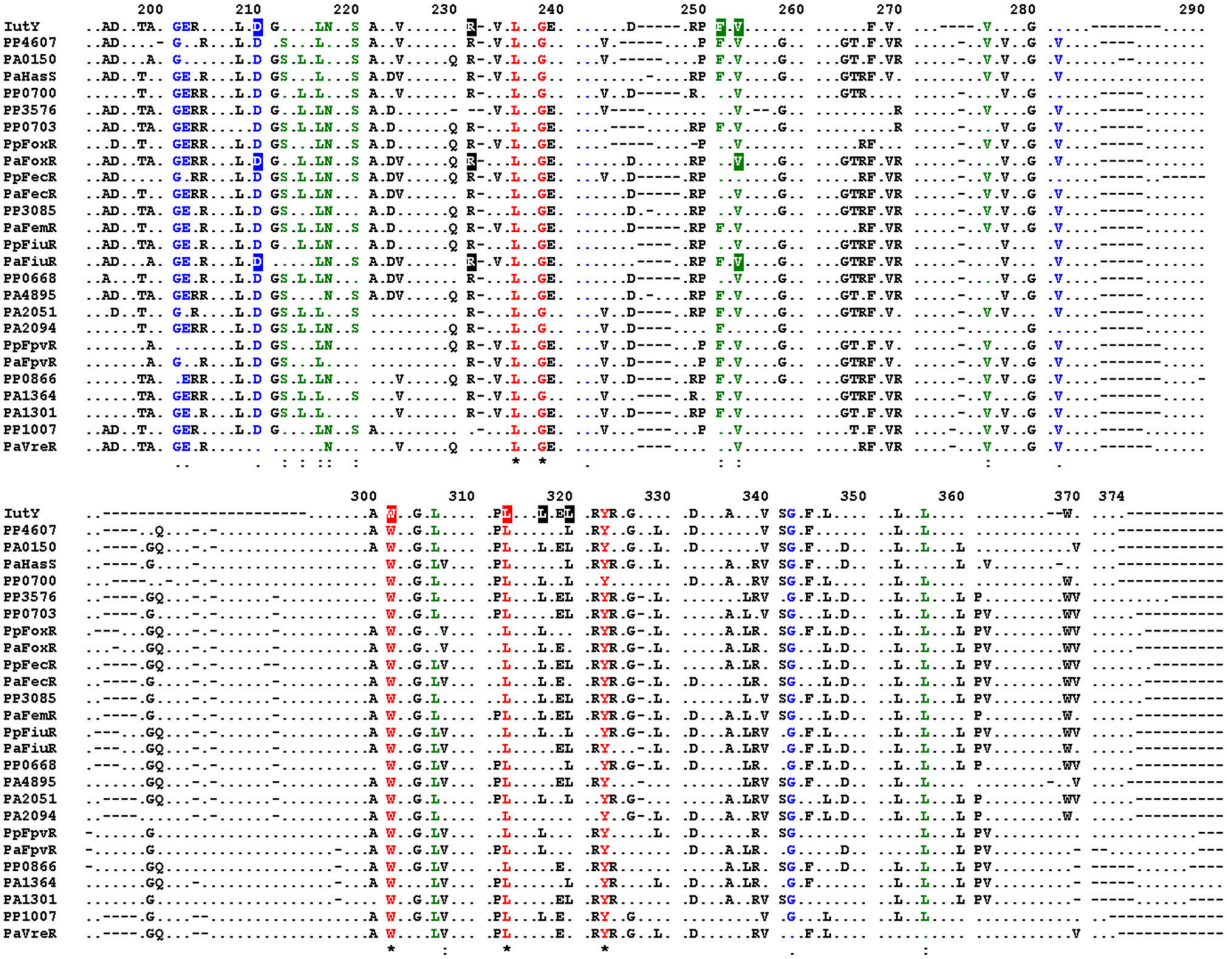
**Conserved residues in ***P. aeruginosa*** and ***P. putida*** CSS anti-sigma factors**. Alignment of the amino acid sequences of the periplasmic regions of all *P. putida* and *P. aeruginosa* CSS anti-sigma factors. Only residues identical in at least 50% of the sequences are shown. Fully conserved residues are shown in red, residues with strong similarity in green, and with weak similarity in blue. Conserved amino acids of which mutation in the *P. putida* IutY protein leads to constitutive activity are boxed, as well as amino acids that were mutated in the *P. aeruginosa* FoxR and FiuR proteins. Numbering indicates amino acid position in the *P. putida* IutY protein.

**Figure 6:**
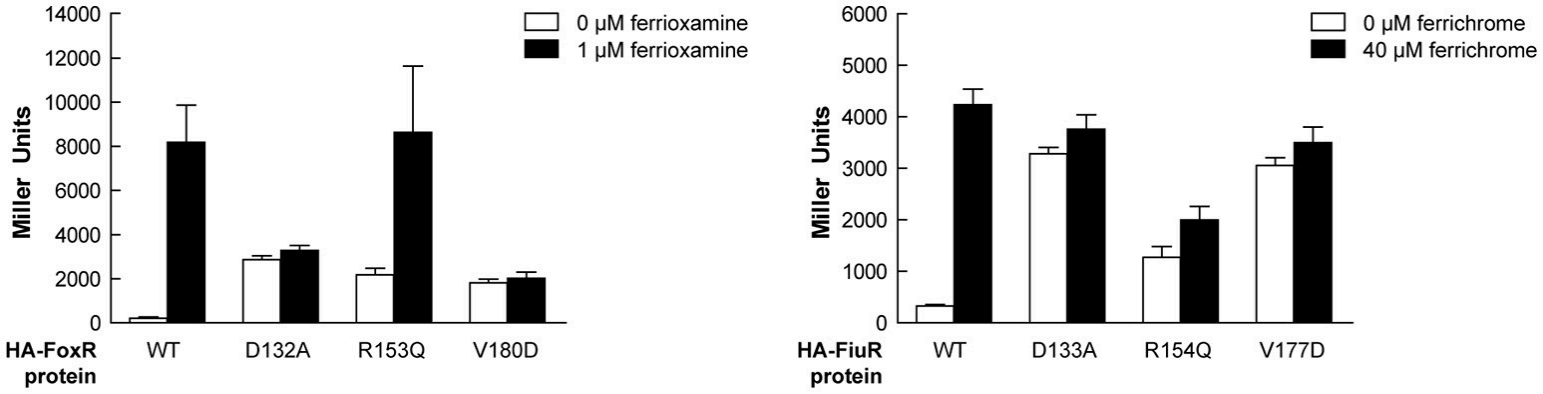
**Activity of the ***P. aeruginosa*** FoxR and FiuR point mutant proteins**. β-Galactosidase activity of *P. aeruginosa* Δ*foxR* bearing the transcriptional fusion *foxA*::*lacZ* (pMPR8b plasmid; left panel) or *P. aeruginosa* Δ*fiuR* bearing the transcriptional fusion *fiuA*::*lacZ* (pMPFiuA plasmid; right panel) and the pMMB67EH-derivate expressing the indicated HA-FoxR or HA-FiuR protein. Strains were grown in low iron medium in the absence (white bars) or presence (black bars) of 1 μM ferrioxamine or 40 μM ferrichrome, respectively.

Alignment of the periplasmic regions of all *P. putida* and *P. aeruginosa* CSS anti-sigma factors (Llamas et al., 2008, 2014) showed that several of the constitutive mutations described above were altering conserved residues (i.e., Asp-210, Arg-231, Phe-251, Val-253, Trp-301, and Leu-313/317/320; Figure 5, boxed residues). To examine whether similar amino acid substitutions in other CSS anti-sigma factors could also induce signal-independent activity of their cognate σ^ECF^, we introduced point mutations in the FoxR and FiuR proteins of *P. aeruginosa* (Figure 5, boxed residues). These *P. aeruginosa* anti-sigma factors are part of classical CSS pathways in which the sigma and the anti-sigma factor proteins are produced as two separate proteins and control the activity of σ^FoxI^ and σ^FiuI^ in response to the siderophores ferrioxamine and ferrichrome, respectively (Llamas et al., 2006). Single point mutations equivalent to the D210A, R231Q, and V253D substitutions in *P. putida* IutY (Figure 3) were introduced in N-terminally HA-tagged versions of both *P. aeruginosa* FoxR (yielding the HA-FoxR-D132A, -R153Q, and -V180D protein variants) and FiuR (yielding the HA-FiuR-D133A, -R153Q, and -V177D protein variants). Activity of these proteins was assayed by β-galactosidase measurements in *P. aeruginosa* Δ*foxR* and Δ*fiuR* mutant strains (Mettrick and Lamont, 2009) bearing the σ^FoxI^- or σ^FoxI^-dependent transcriptional fusion *foxA*::*lacZ* or *fiuA*::*lacZ*, respectively (Llamas et al., 2006). Expression of the wild-type FoxR or FiuR proteins only allowed σ^ECF^-mediated *lacZ* expression in response to the inducing signal (ferrioxamine or ferrichrome, respectively; Figure 6). Production of all mutant proteins resulted in significantly higher σ^ECF^ activity in the absence of the inducing signal (Figure 6, white bars). One of the mutants, FoxR-R153Q, still responded to ferrioxamine (Figure 6), which indicates that this protein is, in contrast to the other mutant proteins, still able to sense and respond to the presence of the siderophore. Several other mutants, i.e., FoxR-D132A, FoxR-V180D, and FiuR-R154Q, did show constitutive activity, albeit at a lower level as compared to the wild-type protein. These mutants seem to have lost the ability to respond to the inducing signal. Finally, we also identified two mutants, FiuR-D133A and -V117D that induced full σ^FiuI^ activity independent of ferrichrome (Figure 6). This indicates that some of the mutations identified in the IutY system have a crucial and conserved function in signal transduction in CSS anti-sigma factors.

In order to provide more insight into the mutations that cause constitutive activity of σ^IutY^, we generated a structural model of the periplasmic region of *P. putida* IutY (IutYperi) using the Protein Homology/analogy Recognition Engine (Phyre2) server (Kelley and Sternberg, 2009; Figure S3). IutYperi is predicted to fold in two distinct regions (i.e., the N- and C-terminus), which are connected by a stretch of 12 amino acids (Figure S3A). We mapped the point mutations that resulted in constitutive activity of σ^IutY^ (Figure S3A, in blue), which showed that Trp-301 is located in the connecting region between the N- and C-terminus of IutYperi. Asp-210, Arg-231, Ala-240, Phe-251, and Val-253 appear to be clustered in the N-terminal periplasmic domain. Interestingly, most of the mutations, with the exception of Ala-240, are located on the same side of the N-terminal domain of IutY. This part of the N-terminal domain is expected to be facing or in close proximity of the cytoplasmic membrane, since the attachment point of the transmembrane segment is also located on this side of the protein. The Leu-313, -317, and -320 residues are present in a α-helix in the C-terminus and substitution of these three amino acids by prolines in the IutY-LtoP mutant is likely to disrupt the secondary structure of this helix. Subsequently, we created similar models for the periplasmic regions of *P. aeruginosa* FiuR (FiuRperi) and FoxR (FoxRperi; Figures S3B,C). This showed that the overall fold of these proteins is predicted to be similar, with the mutations tested in Figure 3 (IutY) and Figure 6 (FoxR and FiuR) in identical positions.

## Discussion

Regulated intramembrane proteolysis (RIP) is a conserved mechanism in which a transmembrane protein is subjected to several processing steps in order to liberate and activate a cytosolic effector (Brown et al., 2000). Recently, we and others have shown that activation of CSS σ^ECF^ in response to the inducing signal requires RIP of the cognate inhibitor (the anti-sigma factor) (Draper et al., 2011; Bastiaansen et al., 2014, 2015a). This proteolytic regulation always involves the site-2 metalloprotease RseP but also a site-1 protease that generates the RseP substrate. The main evidence that a site-1 cleavage of CSS anti-sigma factors occurs is the accumulation of a slightly larger fragment than the RseP product in a Δ*rseP* deletion mutant, which we have observed for *P. putida* IutY (Bastiaansen et al., 2014), *P. aeruginosa* FoxR (Bastiaansen et al., 2015a), and *P. aeruginosa* FiuR (unpublished data). In a previous study, we identified the C-terminal processing protease Prc as the protease that mediates the site-1 cleavage of the unique sigma/anti-sigma hybrid protein IutY (Bastiaansen et al., 2014). Based on these results, we proposed a general model for IutY activation that is illustrated in Figure 1. In this study, we have used a random mutagenesis screen of the IutY protein to further examine the proteolytic activation pathway. By analyzing several truncated versions of the 374 amino acids long IutY protein, we have been able to determine that truncated IutY variants shorter than 237 amino acids, and therefore containing only ~50 periplasmic residues, are completely independent of Prc for activation, which suggests that these proteins became direct substrates for the RseP protease. In contrast, truncated IutY variants of 260 amino acids in length or longer require Prc for full activation. Substrate recognition by Prc has been reported to involve the residues located at the final C-terminus of the target protein, where it prefers to bind small hydrophobic amino acids (Silber et al., 1992; Keiler et al., 1995; Keiler and Sauer, 1996). However, Prc is able to process truncated IutY variants that do not contain the final C-terminal residues of the wild-type protein, which implies that other domains of IutY are involved in the recognition by Prc. Our results suggest that processing of IutY by Prc involves cleavage(s) between (at least) residues 259–374 that shortens the periplasmic domain of IutY, thereby likely generating the substrate for RseP. The detection of several IutY N-terminal subproducts of different but conserved length when the IutY protein is activated (by either aerobactin or single mutations, Figures 3, 4) suggests that the site-1 cleavage of IutY involves (or is followed by) trimming of the protein. Although Prc has been implicated in trimming two stress-responsive anti-sigma factors (Reiling et al., 2005; Qiu et al., 2007; Heinrich et al., 2009), this protease does not seem to be responsible of trimming IutY since the IutY sub-products can be also detected in the Δ*prc* mutant (Figure 4, F251S or LtoP proteins). All together these data, combined with the knowledge that Prc is a C-terminus recognizing endoprotease, result in the hypothesis that, upon activation of the Iut system by aerobactin, alterations occur in the C-terminus of IutY that produce the stepwise degradation of IutY until at least amino acid 259. The resulting IutY protein is subsequently cleaved by RseP. RseP also seems to play a role in the stability of IutY, including that of the σ^IutY^ domain (Figure 2D, IutY-168 variant). Cell fractionation experiments indicated that IutY is tightly associated with the membrane, even the cytosolic sigma domain (data not shown), which could occur through the interaction with RseP. Further, analyses are needed to understand this process.

Signal-independent activation by truncation of the anti-sigma factor C-domain of σ^ECF^ belonging to classical CSS systems—in which the sigma and anti-sigma functions reside in two different proteins—has been also reported for *P. aeruginosa* FoxR and FiuR (Llamas et al., 2006) and *E. coli* FecR (Welz and Braun, 1998). The site-1 protease that mediates the cleavage of these proteins has not been identified yet, but it is not Prc. In contrast to the Iut CSS system, inactivation of Prc only decreases but does not abolish activity of the Fox and Fiu CSS systems (Bastiaansen et al., 2014). Moreover, Prc is not required for the site-2 RseP-mediated cleavage of the FoxR and FiuR anti-sigma factors to occur (Bastiaansen et al., 2015a and unpublished). Although not fully understood yet, the role of Prc in the regulation of these classical CSS pathways seems to be related with maintaining correct anti-sigma factor levels (Bastiaansen et al., 2015a).

Importantly, despite differences in the proteolytic regulation of CSS systems, in this study we have identified several conserved residues of CSS anti-sigma factors of which single substitution leads to activation of their cognate σ^ECF^ independent of the presence of the inducing signal. Therefore, signal-independent activation of σ^ECF^ by small modifications of the periplasmic domain of the anti-sigma factor seems to be a general phenomenon for CSS systems. All IutY full-length mutant variants with constitutive σ^IutY^ activity we tested depended on the site-2 protease RseP for activation, which indicates that they were correctly inserted in the membrane. Interestingly, Prc dependency differed amongst these mutant proteins. However, the fact that most proteins are processed by Prc in the absence of aerobactin (Figure 3) indicates that this protease is constitutively active and that all the components required for the activation of the σ^IutY^ domain are functional in non-inducing conditions. Thus, the sensor for σ^IutY^ activation is not the site-1 protease, unlike for other σ^ECFs^ that are regulated by stress response systems lacking an outer membrane receptor (Ellermeier and Losick, 2006; Qiu et al., 2007; Ades, 2008). Then how does the Prc-dependent cleavage of IutY start *in vivo* in response to aerobactin? We have reported previously (Bastiaansen et al., 2014) and confirmed here that overexpression of the wild-type IutY protein results in an increase of σ^IutY^ activity in the absence of aerobactin, which implies that a negatively regulating element is titrated. Perhaps an extra component binds the periplasmic C-domain of IutY in the absence of aerobactin protecting it from degradation. This mechanism has been described for the control of the *E. coli* stress responsive sigma factor σ^E^, in which the RseB protein binds to the anti-sigma factor RseA and prevents site-1 cleavage (Cezairliyan and Sauer, 2007; Kim et al., 2010). RseB is however not involved in the Iut pathway since overexpression of this protein in *P. putida* had no effect on the activity of this system (data not shown). It is possible that point mutations in IutY disrupt the binding of this hypothetical protein thereby making the periplasmic anti-sigma domain susceptible to proteolytic cleavage even in non-inducing conditions. However, several attempts to identify this putative protecting protein by pull-down experiments did not yield significant results (data not shown). The Asp-210, Arg-231, Phe-251, and Val-253 residues are all located on the same side of the N-terminus region of the periplasmic domain of IutY (IutYperi; Figure S3), which suggests that they form an interaction interface. Interestingly, this part of the protein is probably located very close to the cytoplasmic membrane and therefore does not contact the outer membrane receptor. Furthermore, it has been proposed that Prc prefers to cleave substrates that are not tightly folded (Keiler et al., 1995; Keiler and Sauer, 1996). Thus, mutations in the periplasmic anti-sigma domain of IutY might also result in conformational changes that expose the Prc cleavage sites. In agreement with this, the IutY-R231Q, -W301G, and -LtoP mutant proteins depend on Prc for full activation. In contrast, the IutY-F251S and -V253D proteins were almost completely independent of Prc. These two last IutY variants could have become direct substrates for the site-2 RseP protease, thereby bypassing the requirement for Prc cleavage. Alternatively, these mutations might render the protein susceptible to (an)other periplasmic protease(s) that generate the RseP substrate. Although it was originally proposed that RseP substrate recognition and cleavage was based on the presence of a C-terminal hydrophobic amino acid (Li et al., 2009), recent results have shown that substrate discrimination occurs through the size-filtering function of the two PDZ domains of RseP, rather than by the recognition of a specific sequence/motif (Hizukuri et al., 2014). This suggests that the Prc-independent IutY-F251S and -V253D full-length mutant proteins need to be processed by (an)other protease(s) prior to RseP cleavage, and we are currently trying to identify such protease. Strikingly, our data indicate that IutY protein variants that are not (fully) active are not stable and degraded in a manner in which the cytosolic σ^IutY^ domain is not activated. The mechanism and the protease(s) responsible for this are currently unclear, but this implies that proteolytic regulation of σ^ECF^ activity is even more complex than previously anticipated.

None of the IutY full-length mutant proteins we have analyzed depended on the IutA receptor for activation. In the current CSS model, perception of the CSS signal by the receptor promotes the interaction between the signaling domain of the receptor and the anti-sigma factor in the periplasm (Figure 1). This model is mainly based on experiments with the *E. coli* Fec system that showed that introduction of mutations that compromises the interaction between the FecA receptor and the FecR anti-sigma factor reduce the activity of σ^FecI^ in response to the inducing signal ferric citrate (Enz et al., 2003; Breidenstein et al., 2006). Among the mutations described were those in the FecR leucine-rich motif, which is a conserved motif in the periplasmic region of anti-sigma factors (Figure 5 and Enz et al., 2003). However, mutating this domain of the *P. putida* IutY protein (LtoP mutant variant) did not abolish CSS activity, and, in contrast, the mutation resulted in constitutive activity of the σ^IutY^ domain. This indicates that the IutA-IutY interaction can be impaired without inhibiting the sigma factor activity, and suggests that the CSS receptor could function in a different way as initially thought. The signaling domain of CSS receptors display a common fold, in which two α-helices are flanked by two β-sheets (Garcia-Herrero and Vogel, 2005; Wirth et al., 2007). The region that connects the signaling domain to the outer membrane-located β-barrel domain of the receptor is long and flexible and has been proposed to enable movement of the signaling domain in the periplasm upon signal perception, thereby promoting the interaction with the anti-sigma factor. However, although the orientation of the signaling domain changes in response to the inducing signal, signal perception does not induce alterations in its overall structure and this domain does not extend further into the periplasm (Wirth et al., 2007; Mokdad et al., 2012). This suggests that an interaction between the signaling domain and the anti-sigma factor might already occur in the absence of the inducing signal. This is supported by biochemical interaction studies with the *E. coli* FecA and FecR proteins performed in the absence of the inducer (Enz et al., 2000). Moreover, whereas overexpression of the FecA signaling domain inhibits activity of σ^FecI^ in strains containing a wild-type FecR protein, it does not affect the constitutive activity caused by C-terminally truncated FecR derivatives (Kim et al., 1997), which suggests that this domain binds to the periplasmic domain of FecR. Likewise, overexpression of the *P. putida* IutA signaling domain completely inhibits σ^IutY^ activity in inducing conditions (unpublished results). These data indicate that only the binding of the signaling domain to the anti-sigma factor is not sufficient to trigger activity of the CSS pathway and suggest that this interaction can protect the anti-sigma factor from downstream proteolytic degradation. This introduces an additional level of complexity in the CSS signal transduction cascade and shows that the signaling domain of the receptor deserves more attention in future research.

## Author contributions

KB, WB, and ML conceived and designed the study. KB and CC performed the experiments. KB, WB, and ML analyzed and interpreted the data, and wrote the manuscript.

## Funding

This work was supported by the EU Seventh Framework through a Marie Curie CIG grant (3038130), the Netherlands Organization for Scientific Research (NWO) through an ECHO grant (2951201), and the Spanish Ministry of Economy with grants inside the Ramon&Cajal (RYC2011-08874) and the Plan Nacional for I+D+i (SAF2012-31919 and SAF2015-68873-P) programs.

### Conflict of interest statement

The authors declare that the research was conducted in the absence of any commercial or financial relationships that could be construed as a potential conflict of interest.

## Abbreviations

CSS: cell-surface signaling
ECF: extracytoplasmic function
IPTG: isopropyl β-D-1-thiogalactopyranoside
RIP: regulated intramembrane proteolysis
RNAPc: RNA polymerase core enzyme.
