# Supplementary material for: New Insights into the Regulation of Cell-Surface Signaling Activity Acquired from a Mutagenesis Screen of the Pseudomonas putida IutY Sigma/Anti-Sigma Factor
Source: Front Microbiol. 2017 May 2;8:747. doi: 10.3389/fmicb.2017.00747 (PMC5411451; doi:10.3389/fmicb.2017.00747)
Supplement: Supplementary file 1 [file DataSheet1.PDF]

Supplementary Material

New insights into the regulation of cell-surface signaling activity acquired from a mutagenesis screen of the *Pseudomonas putida* IutY sigma/anti-sigma factor

Karlijn C. Bastiaansen, Cristina Civantos, Wilbert Bitter, and María A. Llamas*

*** Correspondence:** Dr. María A. Llamas, marian.llamas@eez.csic.es

Figure S1

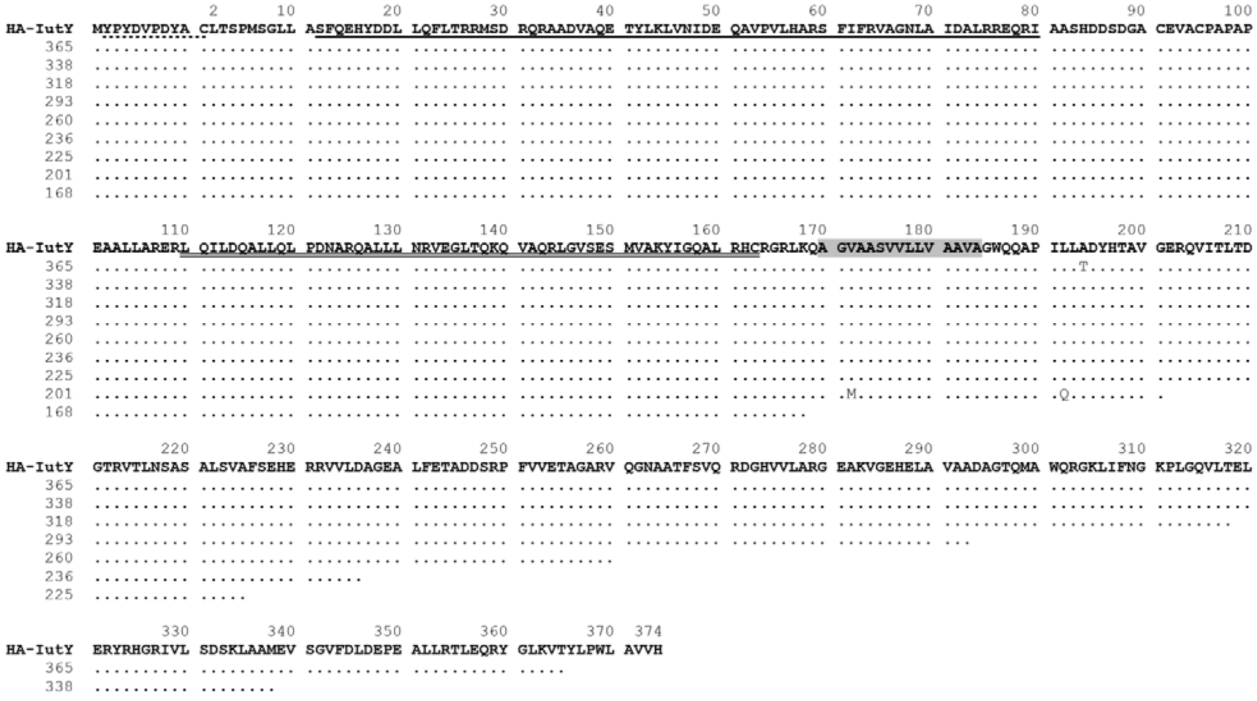

Fig. S1. Truncated IutY variants used for further analysis. Alignment of the amino acid sequence of the N-terminally HA-tagged *P. putida* IutY protein (HA-IutY) with the truncated mutants analyzed in this study. The HA-tag (striped underlined), the σ_2 (underlined) and σ_4 (double underlined) structural domains of σ^{ECFs}, and the transmembrane segment (highlighted grey) are indicated.

Figure S2

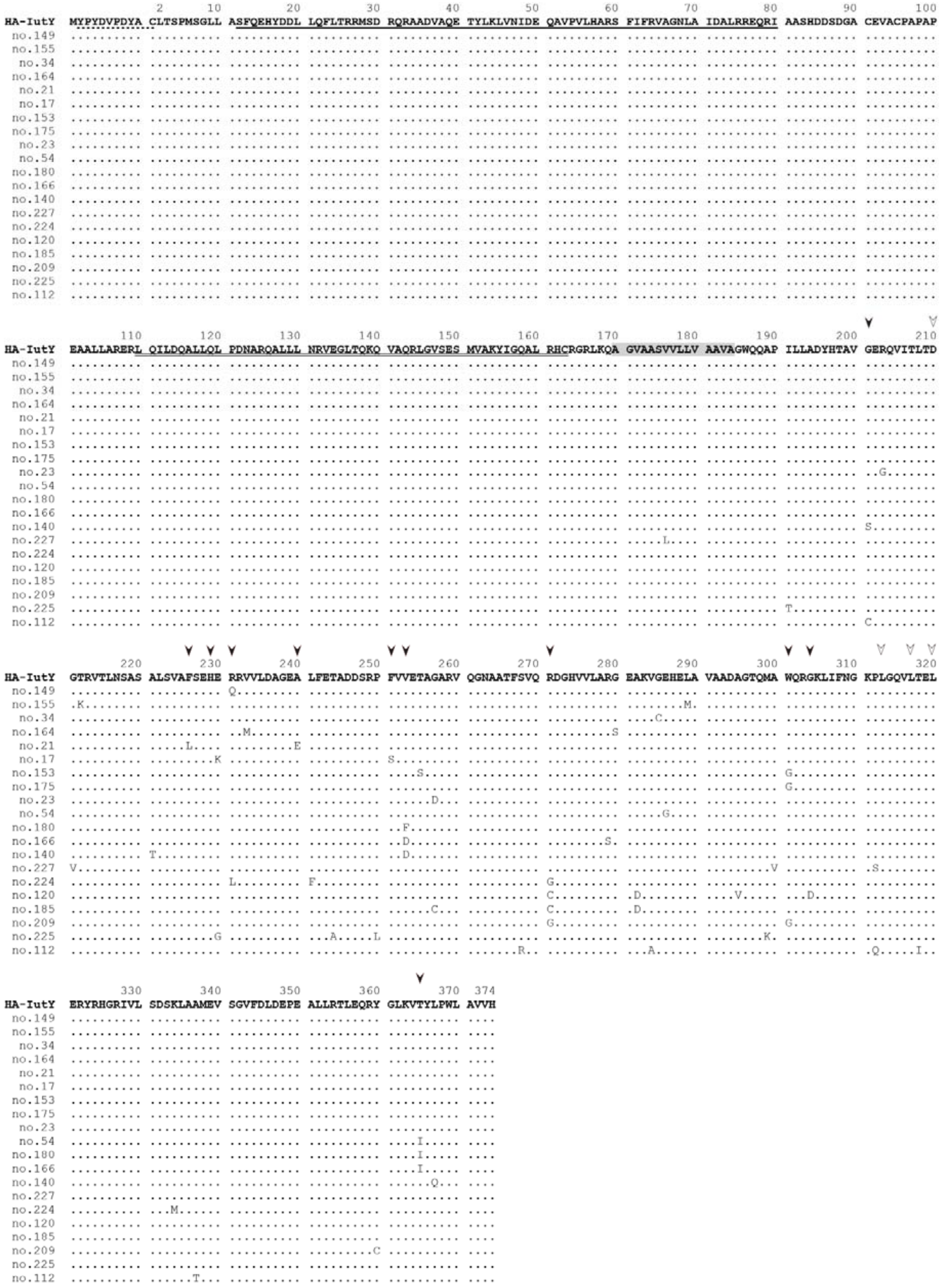
2 10 20 30 40 50 60 70 80 90 100
HA-IutY MYPYDVPDYA CLTSPMSGLL ASFQEHYDDL LQFLTRRMSD RQRAADVAQE TYLKLVNIDE QAVPVLHARS FIFRVAGNLA IDALRREQRI AASHDDSDGA CEVACPAPAP
110 120 130 140 150 160 170 180 190 200 210
HA-IutY EAALLARERL QILDQALLQL PDNARQALLL NRVEGLTQKQ VAQRLGVSES MVAKYIGQAL RHCRGRLKQA GVAASVVLLV AAVAGWQQAP ILLADYHTAV GERQVITLTD
220 230 240 250 260 270 280 290 300 310 320
HA-IutY GTRVTLNSAS ALSVAFSEHE RRVVLDAGEA LFETADDSRP FVVETAGARV QGNAATFSVQ RDGHVVLARG EAKVGEHELA VAADAGTQMA WQRGKLIFNG KPLGQVLTEL
330 340 350 360 370 374
HA-IutY ERYRHGRIVL SDSKLAAMEV SGVFDLDEPE ALLRTLEQRY GLKVTYLPWL AVVH
no.149
no.155
no.34
no.164
no.21
no.17
no.153
no.175
no.23
no.54
no.180
no.166
no.140
no.227
no.224
no.120
no.185
no.209
no.225
no.112

Fig. S2. Full-length IutY point mutants. Alignment of the amino acid sequence of the N-terminally HA-tagged *P. putida* IutY protein (HA-IutY) with the full-length point mutants identified in the random mutagenesis screen. The single mutations introduced in the *iutY* gene are indicated with an arrow above the sequence of HA-IutY (black; mutations derived from screen, white; extra mutations introduced). The HA-tag (striped underlined), the σ_2 (underlined) and σ_4 (double underlined) structural domains of σ^{ECFs}, and the transmembrane segment (highlighted grey) are indicated.

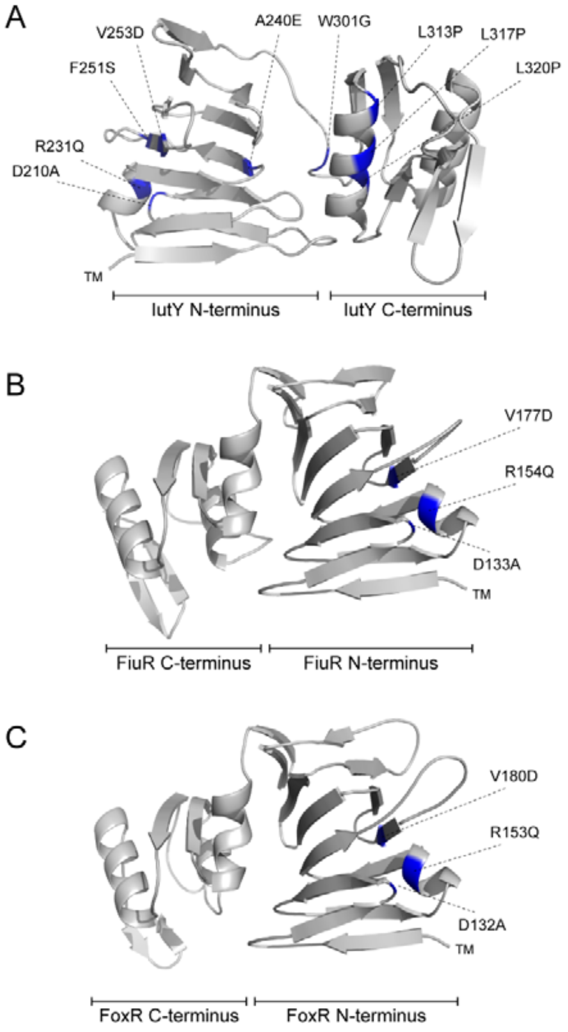

Figure S3. Structural model of the periplasmic region of IutY, FiuR and FoxR. (A) A cartoon representation of the structural model of the *P. putida* IutY periplasmic region created using the Phyre program (Kelley and Sternberg, 2009) is drawn. In the predicted model 100% of the sequence was assigned, based on the crystal structure of the putative anti-sigma factor BDI_1681 from *Parabacteroides distasonis* ATCC8503 (Protein Data Bank accession number 4M0H). We used the DALI (Holm and Rosenstrom, 2010) and TM-align (Zhang and Skolnick, 2005) servers to assess the quality of the IutYperi model. **(B and C)** Cartoon representation of the structural model of the periplasmic region of *P. aeruginosa* FoxR (B) and *P. aeruginosa* FiuR (C) using Phyre and the same structural template (Bastiaansen et al., 2015b). Mutations that resulted in activity of the cognate cytosolic σ^{ECF} (domain) in the absence of the inducing CSS signal are indicated in blue. The two distinctly folding domains of the periplasmic regions of the anti-sigma factors (e.g. the N- and C-terminus) are defined below the cartoon. To allow better visualization of the mutations, the models of FiuRperi and FoxRperi are rotated 180 degrees compared with the model of IutYperi.

Table S1. Sequence of the primers used in this study

Gene (or promoter region)	Plasmid	Primer name	Primer sequence (5’→ 3’)[a]
***P. putida* KT2440**			
iutY (PP2192)	pMMBK1-HA-225	NHA-PP2192-E	AAA**GAATTC**ATGTACCCGTACGACGTGCCGGACTACGCGTGCCTGACTTCACCCATGTCGGGCC
		PP_2192R-H(4b)	CAC**AAGCTT**CACGCCACGCTCAAGGCACTGG
	pMMBK1-HA-236	NHA-PP2192-E	AAA**GAATTC**ATGTACCCGTACGACGTGCCGGACTACGCGTGCCTGACTTCACCCATGTCGGGCC
		PP_2192R-H(4)	CAC**AAGCTT**CAATCGAGTACCACACGCCGT
	pMMBK1-HA-293	NHA-PP2192-E	AAA**GAATTC**ATGTACCCGTACGACGTGCCGGACTACGCGTGCCTGACTTCACCCATGTCGGGCC
		PP_2192R-H(5b)	TAC**AAGCTT**CAGGCCGCCACTGCCAGTTCATG
	pMMBK1-HA-338	NHA-PP2192-E	AAA**GAATTC**ATGTACCCGTACGACGTGCCGGACTACGCGTGCCTGACTTCACCCATGTCGGGCC
		PP_2192R-H(6)	TGT**AAGCTT**CACATCGCAGCCAGCTTGCTGTCG
	pMMBK1-HA-G201C	NHA-PP2192-E	AAA**GAATTC**ATGTACCCGTACGACGTGCCGGACTACGCGTGCCTGACTTCACCCATGTCGGGCC
		IutYR-G201C	ATGACCTGCCGCTCGCACACGGC
		IutYF-G201C	CCACACCGCCGTGTGCGAGCGGC
		PP2192R-X	TTT**TCTAGA**TCAATGCACCACGGCCAACCACGGCAG
	pMMBK1-HA-D210A	NHA-PP2192-E	AAA**GAATTC**ATGTACCCGTACGACGTGCCGGACTACGCGTGCCTGACTTCACCCATGTCGGGCC
		IutYR-D210A	CCCGTGTGCCAGCTGTCAGG
		IutYF-D210A	CATTACCCTGACAGCTGGCAC
		PP2192R-X	TTT**TCTAGA**TCAATGCACCACGGCCAACCACGGCAG
	pMMBK1-HA-F226L	NHA-PP2192-E	AAA**GAATTC**ATGTACCCGTACGACGTGCCGGACTACGCGTGCCTGACTTCACCCATGTCGGGCC
		IutYR-F226L	CACGCCGTTCATGCTCGCTTAACGC
		IutYF-F226L	AGCGTGGCGTTAAGCGAGCATGAAC
		PP2192R-X	TTT**TCTAGA**TCAATGCACCACGGCCAACCACGGCAG
	pMMBK1-HA-E230K	NHA-PP2192-E	AAA**GAATTC**ATGTACCCGTACGACGTGCCGGACTACGCGTGCCTGACTTCACCCATGTCGGGCC
		IutYR-E230K	CGAGTACCACACGCCGTTTATGCTC
		IutYF-E230K	CGTTCAGCGAGCATAAACGGCGT
		PP2192R-X	TTT**TCTAGA**TCAATGCACCACGGCCAACCACGGCAG
	pMMBK1-HA-R231Q	NHA-PP2192-E	AAA**GAATTC**ATGTACCCGTACGACGTGCCGGACTACGCGTGCCTGACTTCACCCATGTCGGGCC
		IutYR-R231Q	CGAGTACCACACGCTGTTCATG
		IutYF-R231Q	CAGCGAGCATGAACAGCGTG
		PP2192R-X	TTT**TCTAGA**TCAATGCACCACGGCCAACCACGGCAG
	pMMBK1-	NHA-PP2192-E	AAA**GAATTC**ATGTACCCGTACGACGTGCCGGACTA

	HA-A240E		CGCGTGCCTGACTTCACCCATGTCGGGCC
		IutYR-A240E	CGGCGGTCTCGAACAACTCCTCAC
		IutYF-A240E	ATGCGGGTGAGGAGTTGTTCGAG
		PP2192R-X	TTT**TCTAGA**TCAATGCACCACGGCCAACCACGGCAG
	pMMBK1-HA-F251S	NHA-PP2192-E	AAA**GAATTC**ATGTACCCGTACGACGTGCCGGACTACGCGTGCCTGACTTCACCCATGTCGGGCC
		IutYR-F251S	CACCGGCCGTTTCGACCACAGACGG
		IutYF-F251S	TTCACGTCCGTCTGTGGTCGAAAC
		PP2192R-X	TTT**TCTAGA**TCAATGCACCACGGCCAACCACGGCAG
	pMMBK1-HA-V253D	NHA-PP2192-E	AAA**GAATTC**ATGTACCCGTACGACGTGCCGGACTACGCGTGCCTGACTTCACCCATGTCGGGCC
		IutYR-V253D	CACCGGCCGTTTCGTCCACAA
		IutYF-V253D	CCGTTTGTGGACGAAACGGCC
		PP2192R-X	TTT**TCTAGA**TCAATGCACCACGGCCAACCACGGCAG
	pMMBK1-HA-R271C	NHA-PP2192-E	AAA**GAATTC**ATGTACCCGTACGACGTGCCGGACTACGCGTGCCTGACTTCACCCATGTCGGGCC
		IutYR-R271C	CGACGTGGCCATCGCACTGC
		IutYF-R271C	CAGCGTGCAGTGCGATGGCC
		PP2192R-X	TTT**TCTAGA**TCAATGCACCACGGCCAACCACGGCAG
	pMMBK1-HA-W301G	NHA-PP2192-E	AAA**GAATTC**ATGTACCCGTACGACGTGCCGGACTACGCGTGCCTGACTTCACCCATGTCGGGCC
		IutYR-W301G	CAGCTTGCCACGCTGCCCGGCC
		IutYF-W301G	CAGATGGCCGGGCAGCGTGGC
		PP2192R-X	TTT**TCTAGA**TCAATGCACCACGGCCAACCACGGCAG
	pMMBK1-HA-G304D	NHA-PP2192-E	AAA**GAATTC**ATGTACCCGTACGACGTGCCGGACTACGCGTGCCTGACTTCACCCATGTCGGGCC
		IutYR-G304D	CGTTGAAGATCAGCTTGTCACGC
		IutYF-G304D	CTGGCAGCGTGACAAGCTGATC
		PP2192R-X	TTT**TCTAGA**TCAATGCACCACGGCCAACCACGGCAG
	pMMBK1-HA-T365I	NHA-PP2192-E	AAA**GAATTC**ATGTACCCGTACGACGTGCCGGACTACGCGTGCCTGACTTCACCCATGTCGGGCC
		IutYR-T365I	CCACGGCAGGTAAATCACC
		IutYF-T365I	CTGAAGGTGATTTACCTGCC
		PP2192R-X	TTT**TCTAGA**TCAATGCACCACGGCCAACCACGGCAG
	pMMBK1-HA-LtoP	NHA-PP2192-E	AAA**GAATTC**ATGTACCCGTACGACGTGCCGGACTACGCGTGCCTGACTTCACCCATGTCGGGCC
		K1-LtoP-Rov	CGGCTCGGTCGGCACCTGCCCCGGCGG
		K1-LtoP-Fov	CCGGGGCAGGTGCCGACCGAGCCGGAGCG
		PP2192R-X	TTT**TCTAGA**TCAATGCACCACGGCCAACCACGGCAG
***P. aeruginosa* PAO1**			
fiuR (PA0471)	pMMB/HA-FiuR-D133A	NHA-PA0471-E	AAA**GAATTC**ATGTACCCGTACGACGTGCCGGACTACGCGTGCAGCGCCGACGATCGTCATTCGC

		PA0471R-D133A	GAGTGGCAGCCGGGAGGG
		PA0471F-D133A	CTGACCCTCCCGGCTGCCACTC
		PA0471Ra-X	TTT**TCTAGA**TCAGCGTGGCACCACTTCGAACC
	pMMB/HA-FiuR-R154Q	NHA-PA0471-E	AAA**GAATTC**ATGTACCCGTACGACGTGCCGGACTACGCGTGCAGCGCCGACGATCGTCATTCGC
		PA0471R-R154Q	TAGCACGACCTGTTGGCGTTC
		PA0471F-R154Q	CGGCGAACGCCAACAGGTC
		PA0471Ra-X	TTT**TCTAGA**TCAGCGTGGCACCACTTCGAACC
	pMMB/HA-FiuR-V177D	NHA-PA0471-E	AAA**GAATTC**ATGTACCCGTACGACGTGCCGGACTACGCGTGCAGCGCCGACGATCGTCATTCGC
		FiuRR-V177D	CGGCGCTGCTGTCCACGAA
		FiuRF-V177D	CCCTTCGTGGACAGCAGCGC
		PA0471Ra-X	TTT**TCTAGA**TCAGCGTGGCACCACTTCGAACC
foxR (PA2467)	pMMB/HA-FoxR-D132A	NHA-PA2467-E	AAA**GAATTC**ATGTACCCGTACGACGTGCCGGACTACGCGTGCGACGGGACGCGCGGTAGGGTC
		PA2467R-D132A	GCAGGCGGGTGCCGGCGACCAGCTC
		PA2467F-D132A	CGAGCTGGTCGCCGGCACCCGCC
		PA2467Ra-X	TTT**TCTAGA**TCAGGCGGCGACCACCCTCAC
	pMMB/HA-FoxR-R153Q	NHA-PA2467-E	AAA**GAATTC**ATGTACCCGTACGACGTGCCGGACTACGCGTGCGACGGGACGCGCGGTAGGGTC
		PA2467R-R153Q	GGAGGATCAGTTGCTGGCC
		PA2467F-R153Q	CGCCGGCCAGCAACTGATCC
		PA2467Ra-X	TTT**TCTAGA**TCAGGCGGCGACCACCCTCAC
	pMMB/HA-FoxR-V180D	NHA-PA2467-E	AAA**GAATTC**ATGTACCCGTACGACGTGCCGGACTACGCGTGCGACGGGACGCGCGGTAGGGTC
		FoxRR-V180D	CTTGCGCGGTACGATCCCGCAG
		FoxRF-V180D	CCGCTGCGGGATCGTACCGC
		PA2467Ra-X	TTT**TCTAGA**TCAGGCGGCGACCACCCTCAC

[a] The sequences of the restriction sites are indicated in bold and the annealing region is underlined
